# Evaluation of acute effect of light-emitting diode (LED) phototherapy on muscle deoxygenation and pulmonary oxygen uptake kinetics in patients with diabetes mellitus: study protocol for a randomized controlled trial

**DOI:** 10.1186/s13063-015-1093-3

**Published:** 2015-12-15

**Authors:** Cristina de Oliveira Francisco, Thomas Beltrame, Cleber Ferraresi, Nivaldo Antonio Parizotto, Vanderlei Salvador Bagnato, Audrey Borghi Silva, Benedito Galvão Benze, Alberto Porta, Aparecida Maria Catai

**Affiliations:** Department of Physiotherapy, Federal University of São Carlos, Rod. Washington Luís, km 235, 13.565-905 São Carlos, São Paulo Brazil; Faculty of Applied Health Sciences, University of Waterloo, 200 University Ave W, N2L 3G1 Waterloo, Ontario Canada; Wellman Center for Photomedicine, Massachusetts General Hospital - Harvard Medical School, 55 Fruit Street, MA 02114 Boston, Massachusetts USA; São Carlos Institute of Physics, University od São Paulo, Av. Trabalhador São-carlense, 400, 13566-590 São Carlos, São Paulo Brazil; Department of Statistics, Federal University of São Carlos, Rod. Washington Luís, km 235, 13.565-905 São Carlos, São Paulo Brazil; Department of Biomedical Sciences for Health, University of Milan, Via Festa del Perdono 7, 20122 Milan, Italy; Department of Cardiothoracic, Vascular Anesthesia and Intensive Care, IRCCS, Policlinico San Donato, Milan, Italy

**Keywords:** Type 2 diabetes mellitus, phototherapy, light-emitting diode, oxygen uptake, physical exercise, oxygen uptake kinetics

## Abstract

**Background:**

Type 2 diabetes mellitus (DM) is responsible for a significant reduction in the quality of life due to its negative impact on functional capacity. Cardiopulmonary fitness impairment in DM patients has been associated with limited tissue oxygenation. Phototherapy is widely utilized to treat several disorders due to expected light-tissue interaction. This type of therapy may help to improve muscular oxygenation, thereby increasing aerobic fitness and functional capacity.

**Methods/Design:**

This study is a randomized, double-blind, placebo-controlled crossover trial approved by the Ethics Committee of the Federal University of São Carlos and registered at ClinicalTrials.gov. Four separate tests will be performed to evaluate the acute effect of phototherapy. All participants will receive both interventions in random order: light-emitting diode therapy (LEDT) and placebo, with a minimum 14-day interval between sessions (washout period). Immediately after the intervention, participants will perform moderate constant workload cycling exercise corresponding to 80 % of the pulmonary oxygen uptake $\left({\mathrm{p}\overset{\cdotp }{\mathrm{V}}\mathrm{O}}_2\right)$ during the gas exchange threshold (GET). LEDT will be administered with a multidiode cluster probe (50 GaAIA LEDs, 850 ηm, 75 mW each diode, and 3 J per point) before each exercise session. Pulmonary oxygen uptake, muscle oxygenation, heart rate, and arterial pressure will be measured using a computerized metabolic cart, a near-infrared spectrometer, an electrocardiogram, and a photoplethysmography system, respectively.

**Discussion:**

The main objective of this study is to evaluate the acute effects of muscular pre-conditioning using LED phototherapy on pulmonary oxygen uptake, muscle oxygenation, heart rate, and arterial pressure dynamics during dynamic moderate exercise. We hypothesize that phototherapy may be beneficial to optimize aerobic fitness in the DM population. Data will be published after the study is completed.

**Trial registration:**

Registered at ClinicalTrials.gov under trial number NCT01889784 (date of registration 5 June 2013).

## Background

Type 2 diabetes (DM) is a significant health problem worldwide due to its high prevalence and mortality [1]. It is characterized by hyperglycemia caused by defects in insulin secretion and/or insulin action. DM is associated with many well-known chronic comorbidities and complications that compromise many tissues, especially the blood vessels, heart, and nerves [2].

Individuals with DM have reduced aerobic fitness characterized by lower peak pulmonary oxygen uptake $\left(p\overset{\cdotp }{V}{O}_2\right)$ [3–6]. In addition, $p\overset{\cdotp }{V}{O}_2$ kinetics analysis has been used to characterize $p\overset{\cdotp }{V}{O}_2$ dynamics during submaximal exercise transitions [5, 7]. The $p\overset{\cdotp }{V}{O}_2$ kinetics during exercise transitions are related to the cardiorespiratory system’s ability to offer O_2_ and the capacity of the exercising muscle to utilize this O_2_ [5, 7]. DM patients have delayed $p\overset{\cdotp }{V}{O}_2$ response after the onset of light to moderate exercise [5, 8] and premature muscular fatigue [9] in comparison with a control group. Many potential mechanisms could explain these impaired responses, for example, reduced muscle blood flow [10] and capillary density [11], defects in muscular oxygen diffusion, and lower mitochondrial oxygen utilization [5] and function [9, 12, 13].

Phototherapy is a widely used resource due to its effect on biological tissues, potentially improving muscular efficiency and aerobic fitness [14–18]. Studies with animals have shown enhanced mitochondrial function [19, 20], microcirculation, and tissue oxygenation [21, 22] mediated by phototherapy. Additionally, studies with healthy humans demonstrate improvements in muscular function, fatigue resistance, $p\overset{\cdotp }{V}{O}_2$, and exercise tolerance [17, 23] and decreased concentrations of lactate and muscle damage markers [23–26].

DM patients have an impaired ability to deliver oxygen (O_2_) to the muscle and possibly to use this O_2_ during exercise, which leads to an imbalance between muscle blood flow and $p\overset{\cdotp }{V}{O}_2$ [27], resulting in hampered $p\overset{\cdotp }{V}{O}_2$ dynamics. Therefore, the main aim of this study is to evaluate the acute effects of LED phototherapy on muscle oxygenation and $p\overset{\cdotp }{V}{O}_2$ kinetics in individuals with DM during moderate cycling exercise. We hypothesize that phototherapy will promote therapeutic effects such as improved aerobic fitness, which could ultimately lead to better functional capacity.

## Methods/Design

### Design

This study is a randomized, double-blind, placebo-controlled crossover trial approved by the Ethics Committee of Federal University of São Carlos (number 13573013.1.0000.5504) and registered at ClinicalTrials.gov under Trial Number NCT01889784. This study will be conducted in accordance with the human research standards set out by the National Health Council Resolution 196/96. The subjects will be included in the study after providing written informed consent.

**Fig. 1:**
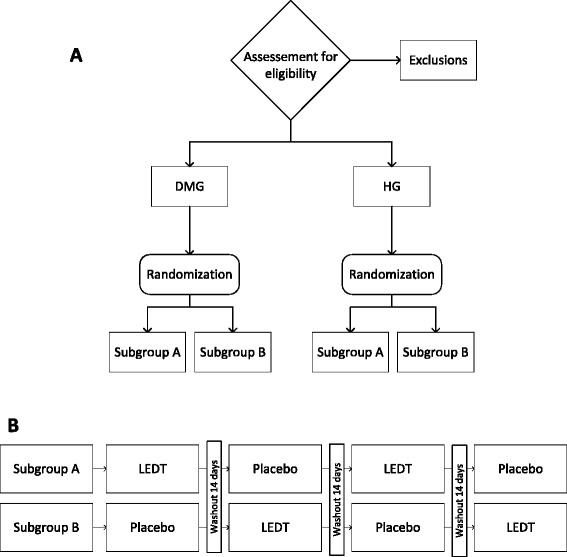
Study design flowchart. Screening and group/subgroup allocation are described in **a**. The intervention order of the study is described in **b**. DMG, diabetes mellitus group; HG, healthy group

The subjects will be divided into two groups: DM group (DMG) and healthy group (HG). After screening, the subjects of both groups who meet the eligibility criteria will be randomly allocated to one of two subgroups (A and B) as described in Fig. 1a. Four separate tests will be conducted to evaluate the acute effect of phototherapy. All subjects will receive both interventions, light-emitting diode therapy (LEDT) and placebo, with a minimum 14-day interval between sessions (washout period). The subgroups will determine the intervention order because the interventions will be alternated in each visit (Fig. 1b). The subjects in subgroup A will receive LEDT in the first and third sessions and placebo in the second and fourth sessions. Subjects in subgroup B will receive the reverse order. The researcher responsible for the randomization process will also prepare the LEDT device (effective or placebo) before each visit and will not analyze the data.

### Subjects

All subjects must be free of overt coronary disease, and they must be between 40 and 64 years of age. DM individuals must have a diagnosis according to the recommendations of the American Diabetes Association [2]. The groups will be matched by age and body mass index (BMI). To achieve that, we will first screen and test the DMG, then evaluate the control group matching age and BMI.

Exclusion criteria are BMI > 35 kg/m^2^, high-sensitivity C-reactive protein (hs-CRP) > 3.0 mg/L, smoking, anemia, alcoholism, use of anti-inflammatory or inhalable drugs, known respiratory and inflammatory diseases, congestive heart failure, and disability conditions that preclude exercise. Healthy subjects will be excluded if they have direct family members with DM.

### Sample characterization

Glycohemoglobin (HbA_1c),_ fasting plasma insulin level, fasting plasma glucose, plasma concentration of CRP, and lipid profile will be measured after 10 to 12 hours of fasting using the analyzer ADVIA 1800 Chemistry System (Siemens, Tarrytown, NY, USA). The degree of insulin resistance will be determined at baseline by the homeostasis model assessment of insulin resistance (HOMA-IR), according to the formula: HOMA-IR = fasting plasma insulin (μU/ml) x fasting plasma glucose (mmol/L)/22.5 [28]. Body composition will be evaluated by tetrapolar bioelectrical impedance analysis (model BC-558, Tanita Corporation of America Inc., Arlington Heights, IL, USA) which calculates body fat, total body water, muscle mass, visceral fat, and bone mass. The subjects will be required to avoid eating and drinking for 4 hours and to urinate prior to the assessment.

### Protocol

**Fig. 2:**
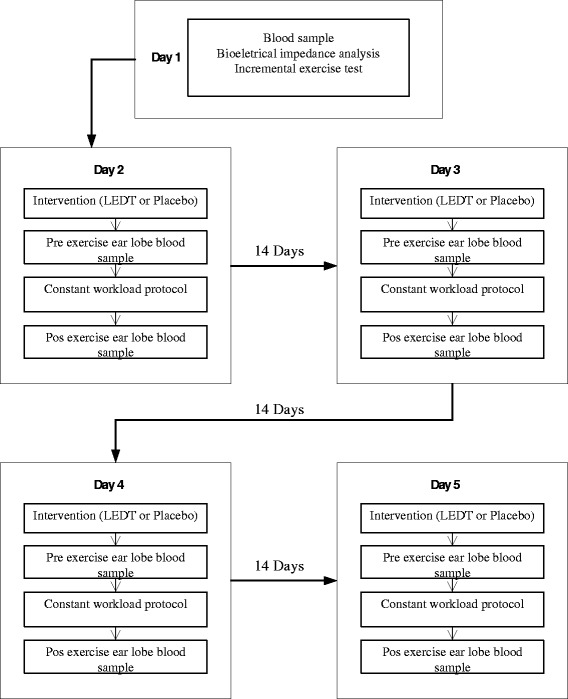
Experimental protocol flowchart. LEDT, light-emitting diode therapy

Following the screening session, the subjects will return to the laboratory on 5 different days (Fig. 2). They will be asked to avoid alcohol and caffeine and refrain from exercise for 24 hours before testing. All tests will take place in the morning, at the same time of the day.

On the first day, incremental exercise on a cycloergometer (Quinton Corival 400, Seattle, WA, USA) will be performed to determine the gas exchange threshold (GET) and peak $p\overset{\cdotp }{V}{O}_2$. Subjects should maintain a cycling cadence of approximately 60 rpm. The workload increment will be determined for each subject in accordance with Wasserman et al. [29]. The GET will be determined by three independent observers according to the ventilatory method [29]. The peak $p\overset{\cdotp }{V}{O}_2$ will be the mean $p\overset{\cdotp }{V}{O}_2$ value of the last 30 seconds during the incremental exercise. It will be assumed that peak $p\overset{\cdotp }{V}{O}_2$ has been achieved when the respiratory exchange ratio (RER) at exercise peak reaches a value higher than 1.1 [30].

After that, the subjects will return to the laboratory four more times to receive the muscular pre-conditioning intervention (LEDT or placebo) according to the randomization. Immediately after the intervention, the subjects will perform moderate constant workload cycling exercise corresponding to 80 % of the $p\overset{\cdotp }{V}{O}_2$ during the GET. The LEDT will be administered with a multidiode cluster probe (50 GaAIA LEDs, 850 ηm, 75 mW each diode, and 3 J per point) before each exercise session. The probe will be in direct contact with skin bilaterally for 40 seconds, delivering 150 J of total energy to each quadriceps and triceps surae muscle. Placebo will follow the same procedure, but, the LEDT device will be turned off.

The constant workload exercise protocol will be preceded by a resting period and 3 minutes of freewheel pedaling. Afterwards, 6 minutes of exercise at target workload will be performed followed by a 6-minute cool-down period of freewheel pedaling.

### Measurements

The $p\overset{\cdotp }{V}{O}_2$, carbon dioxide output (*pVCO*_2_), minute ventilation (VE), and respiratory exchange rate (RER) will be measured breath-by-breath using a computerized metabolic cart (Vmax29c, Sensor Medics, Yorba Linda, CA, USA) previously calibrated for each session.

Electrocardiography (ECG) will be continuously recorded (CM5 lead) with a bioamplifier (BioAmp Power Lab, ADInstruments, Castle Hill, NSW, Australia) and a data acquisition system (Power Lab, ADInstruments, Castle Hill, NSW, Australia). Heart rate (HR) will be estimated based on the ECG signal. Finger arterial pressure will be measured using a photoplethysmography system (Finometer Pro, Finapres Medical System, Amsterdam, Netherlands). The finger cuff will be placed on the middle finger of the left hand. To correct the hydrostatic pressure changes related to the heart, a height correction will be used as recommended by the manufacturer [31].

Local tissue oxygenation of the vastus lateralis and gastrocnemius medialis of the right leg will be measured using near-infrared spectroscopy (NIRS) (Oxymon, Artinis Medical Systems, Nijmegen, Netherlands). NIRS provides measurements of changes in concentrations of deoxygenated and oxygenated hemoglobin (HHb and O_2_Hb, respectively). In addition, the tissue saturation index (TSI) will be measured. To avoid any motion artifact and ambient light influences, the probe will be fixed by tape and then a dark cloth will be gently wrapped around the thigh and calf region.

Before and after the constant workload exercise protocol, blood samples will be collected via earlobe puncture in order to verify lactate and glucose using an automated glucose analyzer (YSI 2300 STAT PLUS – Yellow Springs Instruments, Yellow Springs, OH, USA) that has been previously calibrated according to the manufacturer's instructions.

### Data analysis procedures

The $p\overset{\cdotp }{V}{O}_2$ and NIRS data collected during constant workload exercise will be submitted to time domain kinetics analysis as previously described [5, 32–34]. The data obtained in the two repetitions of the same constant workload protocol for each intervention (LEDT or placebo) will be time-aligned and second-by-second interpolated; then, the point-by-point mean between tests will be finally submitted to kinetics analysis.

### Statistical analysis

The data will be expressed as mean and standard deviation values. The Kolmogorov-Smirnov and Levene tests will be used to test the data for Gaussian distribution and variance, respectively. The statistics analysis will be performed using two-way repeated-measures ANOVA and a post hoc Tukey test for intergroup, intragroup, and multiple comparisons. The level of significance will be set at 5 % (p < 0.05). The statistical power will be set at 0.80, which is the minimal value recommended in the literature to minimize type II error.

## Discussion

The chronic effect of phototherapy has been widely studied and shows promising results related to increased peak $p\overset{\cdotp }{V}{O}_2$, reduction in fatigue, and lactate and C-reactive protein concentrations [23, 24–26]. This novel therapeutic tool is generally studied in healthy participants [17, 18, 23, 25, 26]; however, the acute effects of phototherapy are still unknown in DM and its impact on $p\overset{\cdotp }{V}{O}_2$ dynamics still needs further clarification.

The effects of LEDT could be beneficial to the DM population because the exercise intolerance of this population decreases quality of life and further increases cardiovascular risk. Therefore, the purpose of this randomized clinical trial is to evaluate the acute effects of LEDT on $p\overset{\cdotp }{V}{O}_2$ dynamics in subjects with DM and healthy age- and sex-matched subjects. The results will elucidate the effects of LEDT and may support the use of this therapy in individuals with DM.

Data will be published after the study is completed.

### Trial status

Patient recruitment is currently underway.

## Abbreviations

BMI: body mass index
DM: Type 2 diabetes
DMG: Type 2 diabetes group
ECG: electrocardiogram
GET: gas exchange threshold
HbA_1c_: glycohemoglobin
HHb: deoxygenated hemoglobin
HG: healthy group
(HOMA-IR): homeostasis model assessment of insulin resistance
HR: heart rate
hs-CRP: high-sensitivity C-reactive protein
LED: light emitting diode
LEDT: light-emitting diode therapy
NIRS: near-infrared spectroscopy
O_2_: oxygen
O_2_Hb: oxygenated hemoglobin
pVCO_2_: carbon dioxide output
$\left(p\overset{\cdotp }{V}{O}_2\right)$: pulmonary oxygen uptake
RER: respiratory exchange ratio
TSI: tissue saturation index
VE: minute ventilation
